# Gastrectomy and D2 Lymphadenectomy for Gastric Cancer: A Meta-Analysis Comparing the Harmonic Scalpel to Conventional Techniques

**DOI:** 10.1155/2015/397260

**Published:** 2015-05-14

**Authors:** Hang Cheng, Chia-Wen Hsiao, Jeffrey W. Clymer, Michael L. Schwiers, Bryanna N. Tibensky, Leena Patel, Nicole C. Ferko, Edward Chekan

**Affiliations:** ^1^Ethicon Inc., 4545 Creek Road, Cincinnati, OH 45242, USA; ^2^Cornerstone Research Group, 204-3228 South Service Road., Burlington, ON, Canada L7N 3H8

## Abstract

The ultrasonic Harmonic scalpel has demonstrated clinical and surgical benefits in dissection and coagulation. To evaluate its use in gastrectomy, we conducted a systematic review and meta-analysis of randomized controlled trials comparing the Harmonic scalpel to conventional techniques in gastrectomy for patients with gastric cancer. International databases were searched without language restrictions for comparisons in open or laparoscopic gastrectomy and lymphadenectomy. The meta-analysis used a random-effects model for all outcomes; continuous variables were analyzed for mean differences and dichotomous variables were analyzed for risk ratios. Sensitivity analyses were conducted for study quality, type of conventional technique, and imputation of study results. Ten studies (*N* = 935) met the inclusion criteria. Compared with conventional hemostatic techniques, the Harmonic scalpel demonstrated significant reductions in operating time (−27.5 min; *P* < 0.001), intraoperative blood loss (−93.2 mL; *P* < 0.001), and drainage volume (−138.8 mL; *P* < 0.001). Results were numerically higher for conventional techniques for hospital length of stay, complication risk, and transfusions but did not reach statistical significance. Results remained robust to sensitivity analyses. This meta-analysis demonstrates the clear advantages of using the Harmonic scalpel compared to conventional techniques, with improvements demonstrated across several outcome measures for patients undergoing gastrectomy and lymphadenectomy.

## 1. Introduction

Gastric cancer (GC) is the fourth most frequently occurring cancer in the world, with 989,600 new cases (8% of total) and 738,000 deaths (10% of total) estimated worldwide in 2008 [1, 2]. GC has a significant global burden, with a particularly high incidence in Eastern Europe, South America, and Eastern Asia [2, 3]. Several factors are associated with an increased risk for GC, the most common including* Helicobacter pylori* infection and smoking [4, 5]. Until recently, the most common forms of GC were fundus and distal gastric cancers; however, there has recently been a shift to a greater prevalence of adenocarcinoma [3]. Current treatment guidelines recommend the use of surgical gastric resection for the management of resectable GC; however, there is variation between countries regarding the extent of lymphadenectomy that is performed alongside gastrectomy [1, 3, 6, 7].

Traditionally, hemostasis during gastrectomy has been achieved using a variety of techniques, including suture ligation and monopolar electrosurgery. However, the use of these conventional techniques has been associated with several challenges. For instance, suture ligation not only is a time-consuming process, but is also associated with the risk of knot slipping [8]. Further, in monopolar electrosurgery, the high temperatures (150°C–400°C) that result from using electrical energy to cauterize the tissue can spread into neighboring structures, increasing the risk of injuring surrounding tissue [8, 9].

Ultrasonic devices have been developed to address the challenges associated with conventional hemostasis techniques. Through its design, the Harmonic scalpel simultaneously cuts and coagulates tissue. The device converts electrical energy into mechanical energy, causing the blade to vibrate at a frequency of 55.5 kHz [9]. Simultaneous cutting and coagulation of tissue are achieved by the high frequency vibration of the blade, which generates stress and friction in the tissue molecules. In turn, this disrupts hydrogen bonds and generates heat, causing protein molecules in the tissue to denature and adhere to one another, thereby forming a hemostatic seal. Importantly, no electrical current is passed through the patient, which reduces the risk of burns and injury. Additionally, unlike monopolar electrosurgery, the Harmonic scalpel operates at lower temperatures, thereby dispersing less energy to surrounding tissues and reducing the risk of thermal damage [9, 10].

Several studies have demonstrated the clinical and surgical advantage of the Harmonic scalpel over conventional techniques in different surgical areas, including thyroidectomy [10–13], cholecystectomy [14, 15], and colectomy [16–19]. Furthermore, this device has been widely used in both laparoscopic and open surgeries [10]. More recently, a number of randomized controlled trials (RCTs) have investigated the use of this device compared to conventional techniques in gastrectomy [20, 21]. In this procedure, the Harmonic device is typically used for tissue dissection and sealing of smaller to moderately sized blood and lymphatic vessels, while hemoclips or surgical ties are used for major blood vessels, such as the gastric or gastroepiploic vessels. Results from these studies demonstrate that the Harmonic scalpel can significantly improve outcomes such as operating time in gastrectomy procedures. Further, comparison of ultrasonic devices to conventional methods in a meta-analysis of RCTs and observational studies performed by Chen and colleagues [22] associated beneficial outcomes with ultrasonic scalpels in open gastrectomy procedures.

Presently, there are no meta-analyses examining the exclusive impact of the Harmonic scalpel in gastrectomy. Consequently, this systematic review and meta-analysis paper was conducted to evaluate the performance of the Harmonic scalpel versus conventional techniques in both open and laparoscopic gastric surgery for patients with gastric cancer.

## 2. Methods

A systematic search of 21 databases was conducted, including MEDLINE via PubMed, the Cochrane Central Register of Controlled Trials (CENTRAL), EMBASE, and 18 other national databases (Table 1). Comprehensive searches were additionally conducted using Google Scholar and ResearchGate. Reference lists of retrieved articles were hand-searched. No language restrictions were applied.

Specific inclusion criteria were defined according to PICOS (i.e., population, intervention, comparator, outcomes, and study design). Studies were considered eligible for inclusion if they were RCTs comparing the use of Harmonic surgical devices to conventional methods, such as monopolar electrosurgery and suture, clips, or knot-tying (Table 2), in human subjects, for all types of surgery. Full-text studies were then excluded firstly if they did not focus on either open or laparoscopic gastrectomy for gastric cancer, secondly if they used advanced energy devices other than Harmonic devices, and thirdly if they used the Harmonic device outside of the cleared indication. In the Harmonic studies that were included, the Methods section had to identify that the Harmonic device was the principal device used for the procedures. Records were evaluated for eligibility by two independent reviewers. Disagreements between reviewers regarding study inclusion were resolved through consensus or by consultation with a third reviewer.

For included studies, details (i.e., baseline characteristics and outcomes) were extracted using a comprehensive data extraction form. Data extraction was conducted by two independent reviewers with discrepancies resolved by consensus or a third party. The data from non-English articles were translated, extracted, and included in the form. One reviewer conducted the data extraction, which was cross-checked by a second reviewer.

Clinical outcome measures included the following: (1) operating time, (2) intraoperative blood loss, (3) drainage volume, (4) length of hospitalization, (5) transfusion risk, and (6) complication rate. When necessary, study authors were contacted for additional methodological details regarding whether open or laparoscopic surgery was performed; however, study authors were not contacted for missing data. Missing variance data were calculated from other effect estimates and dispersion measures where feasible and appropriate. Variance measures (i.e., standard deviation (SD)) were not reported in one study [23] for operating time or in another study [24] for drainage volume. Therefore, the missing variance measures were imputed according to the standard methods outlined by Cochrane [25]. Another study reported all continuous outcomes as medians with 95% confidence intervals (CI) [26]; therefore, assuming a normal distribution, the data were imputed using methods outlined by Cochrane [25] and Hozo et al. [27].

The quality of the included studies was assessed using the risk of bias algorithm outlined by the Cochrane guidelines [25]. Studies were scored as having low, unclear, or high risk of bias for the seven domains (sequence generation, allocation concealment, blinding of participants and personnel, blinding of outcome assessment, incomplete outcome data, selective outcome reporting, and other issues) in the assessment tool. Final decisions were dependent on the combination of these factors and the individual characteristics of each study. Study quality was assessed by two independent authors, where disagreements were resolved through consensus or by consultation with a third author.

The meta-analysis was performed using Review Manager (Version 5.3, The Nordic Cochrane Centre, The Cochrane Collaboration, Copenhagen, Denmark, 2014). Continuous variables (operating time, intraoperative blood loss, drainage volume, and length of hospitalization) were analyzed for mean differences (MD) using the inverse-variance method. Dichotomous variables (transfusion risk and complication rate) were analyzed for risk ratios (RR) using the Mantel-Haenszel method. A random-effects model was used for the meta-analysis and forest plots were generated for all outcomes within Review Manager. Heterogeneity of the included studies was assessed using the *χ* ^2^ test and *I* ^2^ measure.

The primary analysis compared the Harmonic scalpel to conventional techniques. Sensitivity analyses were completed for study quality, where studies with unclear or high risk of bias across several measures were excluded [20, 26, 28], and for the type of conventional technique used, where studies using only monopolar electrosurgery (i.e., excluding suture ligation) were included [24, 29–31]. Further, sensitivity analyses were conducted by excluding any study outcomes for which imputed data were required.

## 3. Results

**Figure 1:**
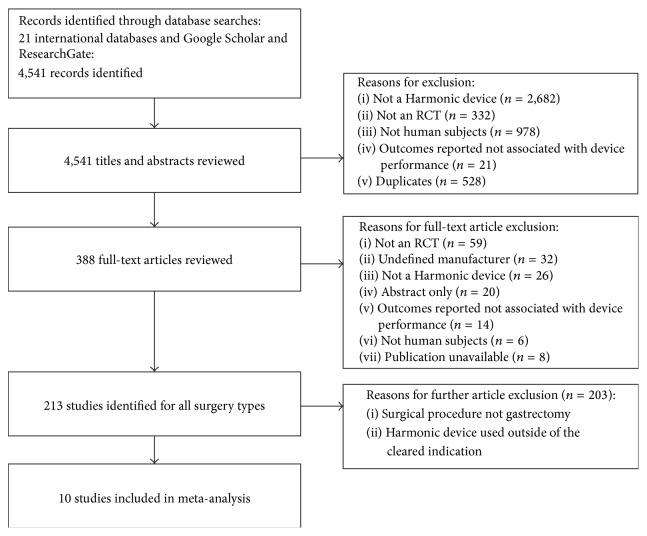
PRISMA diagram for the systematic literature review.

A total of 4,541 records were identified from database searching, of which 4,153 were excluded during the title and abstract review (Figure 1). Of the 388 full-text articles retrieved for review, 378 were further excluded if studies were non-RCTs, had an undefined manufacturer, and did not use a Harmonic device within the cleared indication, the publication was unavailable and had nonhuman subjects, or the surgical procedure was not gastrectomy. Ten studies, consisting of a total of 935 patients that reported on the use of Harmonic devices in gastrectomy, were included in the meta-analysis [20, 21, 23, 24, 26, 28–32].

Study characteristics are presented in Table 2. Sample sizes of the included studies ranged from 40 to 253 patients and study length ranged from 11 to 48 months. In all studies, Harmonic surgical devices (i.e., Harmonic scalpel, Harmonic Wave, and Harmonic Focus) were compared to conventional techniques in gastrectomy. In five studies, the conventional method used was monopolar electrosurgery alone [24, 28–31]; four studies reported using monopolar electrosurgery in combination with suture ligation or clips [21, 23, 26, 32]; one study did not report the specific conventional methods used [20]. All studies performed lymphadenectomy alongside gastrectomy. Of the ten included studies, five were Chinese; however, patient characteristics were comparable across all included studies. Operating time was reported in nine studies [20, 21, 23, 26, 28–32] and eight studies reported intraoperative blood loss [20, 21, 26, 28–32]. One study [24] reported operating time for lymphadenectomy patients only and was therefore excluded from this outcome. Nine studies [20, 21, 24, 26, 28–32] reported on drainage volume and three studies reported the postoperative length of hospital stay [26, 28, 31]. Five included studies described the complication rate for both treatment groups [21, 24, 26, 28, 31] and the rate for blood transfusions was reported in two studies [26, 28]. Of the 10 included studies, nine reported on open gastrectomy and one on laparoscopic gastrectomy.

**Figure 2:**
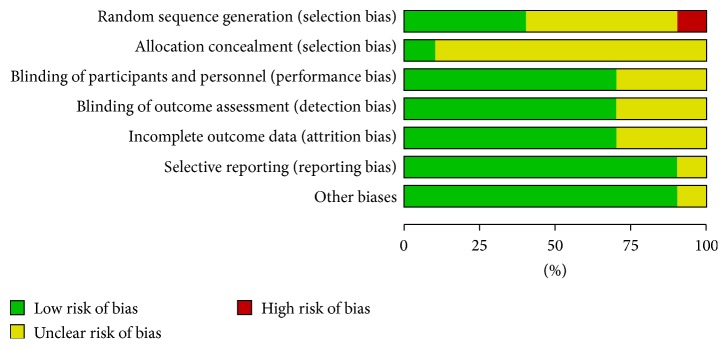
Risk of bias assessment for studies meeting inclusion criteria.

Overall, the risk of bias varied across studies. The overall results of the risk of bias assessments are presented in Figure 2 and the results of the individual study quality assessments are summarized in Table 3. Randomization method was reported in four studies: two studies used a random permuted block design [21, 23], one reported the use of a random number table [31], and one described the use of a lottery system [29]. Only one study [23] described concealment of the randomization sequence. One study [23] reported blinding of patients to the surgical technique. Of the nine studies that did not report blinding, risk of performance bias was deemed low in six studies [20, 21, 24, 29, 30, 32], where nonblinding was assumed to have no impact on the outcomes assessed. Nine studies reported all prespecified study outcomes; however, selective reporting remained unclear in one study [20]. Attritions or exclusions were reported in two studies [21, 23] but were assumed to have no clinical impact on the observed effect size. In three studies, reporting of attritions or exclusions was insufficient [24, 29, 30]. There were no patient withdrawals in five studies [20, 26, 28, 31, 32]. Additional bias was unclear in one study, as there was a reported difference in surgeon status between treatment groups (Harmonic group: 40% residents versus conventional group: 56.7% residents) [26]. Studies generally reported positive results which could result from a publication selection bias; however, funnel plots did not indicate any clear omission of negative studies.

### 3.1. Operating Time

**Figure 3:**
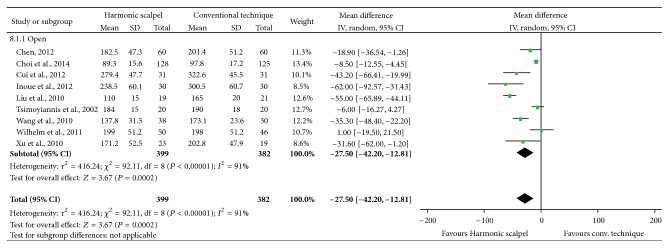
Forest plot of meta-analysis results for operating time (minutes), stratified by open versus laparoscopic surgery.

Mean operating time was statistically significantly reduced by 27.50 minutes (95% CI: −42.20 to −12.81; *P* = 0.0002; 9 studies; *I* ^2^ = 91%) with the Harmonic scalpel in contrast to conventional methods in open gastrectomy (Figure 3). All results were reported for open gastrectomy.

### 3.2. Intraoperative Blood Loss

**Figure 4:**
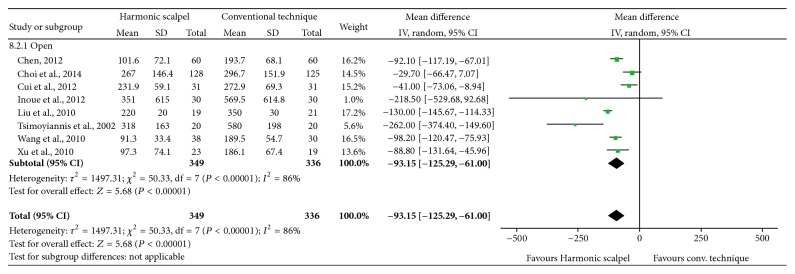
Forest plot of meta-analysis results for intraoperative blood loss (mL), stratified by open versus laparoscopic surgery.

Results demonstrated that, with the Harmonic scalpel, mean intraoperative blood loss was statistically significantly reduced by 93.15 mL (95% CI: −125.29 to −61.00; *P* < 0.00001; 8 studies; *I* ^2^ = 86%) in open gastric resection (Figure 4). All results were reported for open gastrectomy.

### 3.3. Drainage Volume

**Figure 5:**
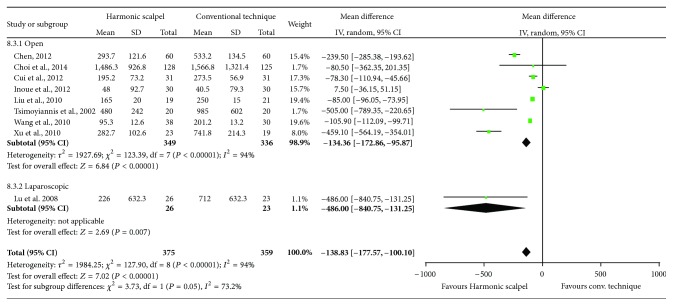
Forest plot of meta-analysis results for drainage volume (mL), stratified by open versus laparoscopic surgery.

Compared to conventional techniques, mean drainage volume was statistically significantly reduced (MD = −138.83 mL; 95% CI: −177.57 to −100.10; *P* < 0.00001; 9 studies; *I* ^2^ = 94%) with the Harmonic scalpel (Figure 5). The study device also showed significant reductions in drainage volume in open surgery (MD = −134.36 mL; 95% CI: −172.86 to −95.87; *P* < 0.00001; 8 studies; *I* ^2^ = 94%). While a subgroup analysis for studies using laparoscopic surgery was not performed due to limited data, the results from the primary analysis and open surgery subgroup analysis were in line with those from the single study that conducted gastrectomy laparoscopically [24].

### 3.4. Length of Hospital Stay

**Figure 6:**
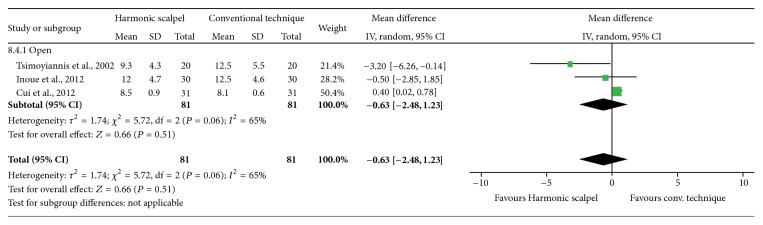
Forest plot of meta-analysis results for length of hospital stay (days).

On the basis of the three studies comparing the Harmonic scalpel to conventional techniques in open gastrectomy, no statistically significant differences in the postoperative length of hospitalization were observed (MD = −0.63 days; 95% CI: −2.48 to 1.23; *P* = 0.51; 3 studies; *I* ^2^ = 65%) (Figure 6). All results were reported for open gastrectomy.

### 3.5. Blood Transfusion

**Figure 7:**
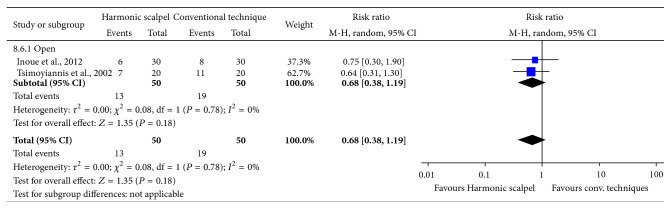
Forest plot of meta-analysis results for patient transfusion risk.

Results demonstrated a lower risk of blood transfusions with the Harmonic scalpel than with conventional methods, although not statistically significant (RR of 0.68; 95% CI: 0.38 to 1.19; *P* = 0.18; 2 studies; *I* ^2^ = 0%) (Figure 7). All results were reported for open gastrectomy.

### 3.6. Postoperative Complications

**Figure 8:**
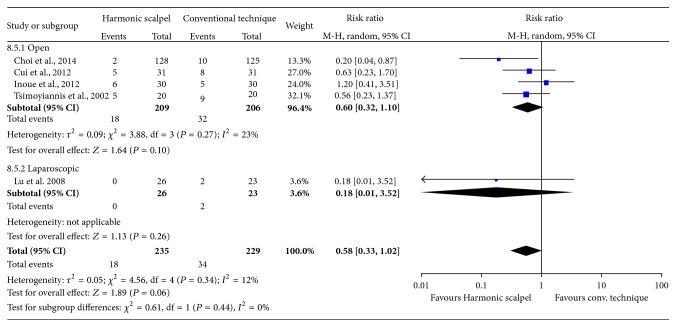
Forest plot of meta-analysis results for total complication rate.

The main reported complications for both study groups included postoperative bleeding, chylous leakage, gastrointestinal paralysis, and wound infection. The Harmonic scalpel reduced the risk of postoperative complications compared to conventional techniques, although not significantly so (RR = 0.58; 95% CI: 0.33 to 1.02; *P* = 0.06; 5 studies; *I* ^2^ = 12%) (Figure 8). Similar results were observed between the open surgery subgroup and the single study reporting on laparoscopic gastric resection.

*Sensitivity Analyses*. Results of sensitivity analyses on study quality were similar to the primary analysis and were relatively robust to variables tested with some exceptions. For operating time, intraoperative blood loss, and drainage volume, results remained statistically significantly lower with the Harmonic scalpel in all sensitivity analyses (Table 4). Also, when studies with a higher risk of bias were removed, it is interesting to note that the Harmonic scalpel significantly reduced the risk of postoperative complications compared to conventional techniques (*P* = 0.03). For hospital length of stay and transfusion risk, there were an inadequate number of studies to conduct a full meta-analysis for some sensitivity variables. Additionally, results of the primary analysis were insensitive to whether or not conventional methods focused solely on monopolar electrosurgery or when the imputed results by Wilhelm et al. [23], Lu et al. [24], and Inoue et al. [26] were excluded for operating time, drainage volume, or all continuous variables, respectively.

## 4. Discussion

Given the challenges associated with conventional hemostatic techniques in gastric surgery, advanced devices that can overcome these drawbacks and improve surgical outcomes may be preferred. This meta-analysis assessed the performance of the Harmonic scalpel compared to electrosurgery or suture ligation in gastrectomy. The breadth of the systematic search was quite extensive, with a large quantity of international, national, and regional databases searched.

The findings of the meta-analysis support the benefit of using the Harmonic scalpel in surgery, with a significant reduction in operating time, drainage volume, and intraoperative blood loss, in comparison to conventional methods. Although no significant differences between groups were observed for other outcome measures, results numerically favored the Harmonic scalpel for reducing length of hospital stay, transfusions, and postoperative complications. The main reported complications for both study groups included postoperative bleeding, chylous leakage, gastrointestinal paralysis, and wound infection. Results for all outcome measures remained relatively consistent when subgroup and sensitivity analyses were conducted, which restricted included studies by type of conventional device, type of surgery, and data imputation of results, highlighting the robustness of the findings. However, sensitivity analyses excluding lower quality studies produced a significant reduction in the risk of complications with the Harmonic scalpel. Methods outlined by Cochrane [25] were used to evaluate the quality and potential bias of the included studies. Results showed that the quality of included studies was acceptable overall, with fairly low risk of bias.

A more general meta-analysis by Chen et al. [22] compared the use of ultrasonic scalpels to conventional techniques in gastrectomy, providing evidence that these devices can reduce operating time and intraoperative blood loss, without compromising patient safety. Chen et al. [22] conducted analyses using both randomized and observational studies. The results of our meta-analysis confirm the findings of this published study, add more recent data, include only RCTs, and exclusively assess Harmonic scalpel use in gastrectomy. Our findings are also aligned with several meta-analyses that evaluated the efficacy and safety of this ultrasonic device in patients undergoing thyroid surgery [10, 12]. In the recent analysis of thyroidectomy by Contin et al. [12], the Harmonic scalpel significantly reduced operating time, blood loss, and postoperative drainage compared to other hemostatic techniques, demonstrating the clear benefits across surgical areas [10]. Furthermore, evidence demonstrates that the favourable outcomes associated with the Harmonic scalpel can translate into cost-savings for hospital institutions [33, 34].

Although all the studies included within this analysis performed gastrectomy plus D2 lymphadenectomy, it is important to note that the extent of lymphadenectomy performed alongside gastric resection differs between countries and institutions [7]. While some suggest that a less extensive D1 lymphadenectomy is the minimum standard for gastric cancer, this is not universally implemented in lower incidence countries (e.g., United States). Conversely, in Eastern Asia, where nearly half of gastric cancer cases occur, D2 lymphadenectomy is typically the standard procedure performed. Prospective studies in both the East and West have shown that less extensive lymphadenectomies likely result in understaging of patients and increased locoregional recurrence; yet the effect on overall survival is more challenging to determine. Further randomized studies in Western patients are required in order to make conclusions regarding the survival benefits of D2 lymphadenectomy.

This study does have limitations. First, not all data were available to inform variance inputs for the meta-analysis. In order to overcome this, imputation methods and assumptions outlined by Cochrane were utilized [25]. Sensitivity analyses excluding outcomes with imputed data showed that results remained similar to the primary analysis. Second, since the heterogeneity between studies was significant for the majority of the meta-analyses, a random-effects model was used to pool all outcome data. Third, data were not available from all studies for each of the included outcomes to be statistically combined in this meta-analysis, and four of the studies had less than 50 subjects. Therefore, some outcome results may not be as rigorous due to small sample sizes. Additionally, not all studies reported the rate of total postoperative complications; therefore the total rates included in the meta-analysis represent an addition of individual complications reported in the studies. Last, because the authors did not have access to the study protocols of the included publications, there is potential for some variation in the study quality. The exclusion of lower quality studies in sensitivity analyses produced a significant reduction in the risk of postoperative complications; however, this exclusion did not impact all other outcomes. Furthermore, this study should be viewed as representative of outcomes for open gastrectomy, since only one of the ten studies was performed laparoscopically. The single laparoscopic study did not evaluate operative time, blood loss, hospital stay, or patient transfusion risk. Exclusion of the laparoscopic study did not have a substantial impact on the parameters that were measured therein, namely, drainage volume and total complication rate.

In summary, the findings of this study hold practical importance. Reductions in blood loss may translate into a lower clinical burden for patients. Relevant to physicians and economic stakeholders, reductions in resource use have potential cost implications for hospitals. For example, reductions in intraoperative blood loss can decrease the need for blood loss management resources, such as expensive blood products, and hemostatic agents. Further, several studies have revealed the high costs associated with operating time [35–37]; hence the use of time-saving devices in surgery can lead to substantial savings. Essentially, products that require fewer people and less steps are more time-cost efficient. These savings in operating time and other resources (e.g., reduced hospital stay) can help to offset the product acquisition cost. However, there is still a need for further costing studies to fully elucidate this impact of resource aversion on potential cost savings.

## 5. Conclusions

The Harmonic scalpel is an effective surgical technique compared to conventional methods in gastrectomy and lymphadenectomy. It offers several clinical advantages, including reduced operating time and blood loss, which can ultimately benefit the surgeon, patient, and hospital, without the addition of safety concerns.

## Figures and Tables

**Table 1:** List of databases and search periods included in systematic search.

Databases	Search dates
EMBASE MEDLINE (via PubMed) CENTRAL	Until 30 September 2013

LILACS and IBECS African Index Medicus, Index Medicus for Eastern and Mediterranean Region, and Index Medicus for South-East Asia Region and The Western Pacific Region Index Medicus African Journals Online IndMed (India) PakMediNet (Pakistan) Türk Tip Veri Tabani (Turkey) Krack (Croatia) SID and IrMedex (Iran) KoreaMed (Korea)	Conducted between 26 and 30 September 2013

ICHUSHI-Web (Japan)	Until 22 April 2013

Wanfang, Cqvip, and CNKI (China)	Until 16 April 2013

**Table 2:** Study and baseline characteristics for studies meeting inclusion criteria.

Reference	Country	Intervention	*n*	Age (mean ± SD)	% male	Surgery	Study length (months)	Included endpoints
Choi et al., 2014 [21]	Korea	Harmonic scalpel Monopolar electric coagulator and clips	128 125	52.8 ± 10.7 53.9 ± 10.5	60.9% 64.8%	Gastrectomy with D2 lymph node dissection	16	(i) Operating time (ii) Intraoperative blood loss (iii) Postoperative lymphatic drainage (iv) Postoperative complication

Chen, 2012 [20]	China	Harmonic scalpel (GEN300) Conventional techniques (knot-tying)	60 60	58.9 ± 19.3	65.0%	Distal radical resection of gastric carcinoma and lymphadenectomy	25	(i) Operation time (ii) Intraoperative blood loss (iii) Drainage volume

Cui et al., 2012 [31]	China	Harmonic scalpel (GEN300) Electrosurgery	31 31	56.4 ± 9.9 58.9 ± 9.2	67.7% 58.1%	Gastrectomy with D2 lymph node dissection	48	(i) Operation time (ii) Blood loss volume (iii) Postoperative drainage volume of the first 72 hours (iv) Length of postoperative hospital stay (v) Complication rate

Inoue et al., 2012 [26]	Japan	Harmonic Focus Electrosurgery and knot-tying	30 30	64 (41–79)^∗^ 67 (45–92)^∗^	70.0% 73.3%	Gastrectomy with lymph node dissections (^∗^combined resection performed in 6 patients in each group)	11	(i) Operative time (ii) Blood loss (iii) Postoperative drainage volume (iv) Postoperative hospital stay (v) Surgery-related complications (vi) Number of transfused patients

Liu et al., 2010 [29]	China	Harmonic scalpel (GEN300) Electrosurgery	19 21	62 64	63.2% 57.1%	Radical distal resection of gastric carcinoma with D2 dissection	29	(i) Operation time (ii) Blood loss (iii) Peritoneal drainage volume of the first 3 days

Lu et al., 2008 [24]	China	Harmonic scalpel (GEN300) Monopolar electrosurgery	26 23	66^∗^ 62^∗^	61.5% 56.5%	Distal radical gastrectomy with D2 dissection^†^	11	(i) Length of operating time for lymphadenectomy (ii) Blood loss in lymphadenectomy (iii) Drainage volume

Tsimoyiannis et al., 2002 [28]	Greece	Harmonic scalpel Monopolar electrosurgery	20 20	59 ± 10 62 ± 9	80% 75%	Gastrectomy with D2 dissection	24	(i) Operative time (ii) Operative blood loss (iii) Postoperative abdominal drainage (iv) Number of patients transfused (v) Postoperative hospital stay (vi) Frequency of major complication

Wang et al., 2010 [30]	China	Harmonic scalpel (GEN300) Monopolar electrosurgery	38 30	61.8 ± 21.5	60.30%	Laparoscopic radical distal D2 gastrectomy	23	(i) Operation time (ii) Intraoperative bleeding volume (iii) Postsurgery 3-day drainage volume

Wilhelm et al., 2011 [23]	Germany	Harmonic Wave Ultrasound Dissector Electrocautery, sutures, and ligatures	100 101	66 ± 12 64 ± 12	60% 61.40%	Left hemicolectomy^‡^ and gastrectomy with D2 lymphadenectomy	24	(i) Duration of operation (ii) Perioperative blood loss (iii) Intraoperative complications (iv) Duration of hospital stay

Xu et al., 2010 [32]	China	Harmonic scalpel Electrosurgery and knot-tying	23 19	61 64	60.90% 63.20%	Distal radical gastrectomy with D2 dissection	18	(i) Operation time (ii) Intraoperative blood loss (iii) Postoperative drainage volume

^∗^Age reported as median (range).

^†^Reported outcomes that included only lymphadenectomy patients were excluded from the analysis.

^‡^Reported outcomes that included patients who received hemicolectomy were excluded from the analysis.

**Table 3:** Qualitative risk of bias assessment summary.

Study	Sequence generation	Allocation concealment	Blinding of personnel and participants	Blinding of outcomes	Incomplete outcome data addressed	Free of selective reporting	Free of other biases
Choi et al., 2014 [21]	Yes	Unclear	Yes	Yes	Yes	Yes	Yes
Chen, 2012 [20]	No	Unclear	Yes	Yes	Yes	Unclear	Yes
Cui et al., 2012 [31]	Yes	Unclear	Unclear	Unclear	Yes	Yes	Yes
Inoue et al., 2012 [26]	Unclear	Unclear	Unclear	Unclear	Yes	Yes	Unclear
Liu et al., 2010 [29]	Yes	Unclear	Yes	Yes	Unclear	Yes	Yes
Lu et al., 2008 [24]	Unclear	Unclear	Yes	Yes	Unclear	Yes	Yes
Tsimoyiannis et al., 2002 [28]	Unclear	Unclear	Unclear	Unclear	Yes	Yes	Yes
Wang et al., 2010 [30]	Unclear	Unclear	Yes	Yes	Unclear	Yes	Yes
Wilhelm et al., 2011 [23]	Yes	Yes	Yes	Yes	Yes	Yes	Yes
Xu et al., 2010 [32]	Unclear	Unclear	Yes	Yes	Yes	Yes	Yes

Yes: low risk of bias; No: high risk of bias.

**Table 4:** Summary of primary and sensitivity analyses.

	Operating time (min) (MD [95% CI])	Intraoperative blood loss (mL) (MD [95% CI])	Drainage volume (mL) (MD [95% CI])	LOS (days) (MD [95% CI])	Complications (*n*) (RR [95% CI])	Patient transfusions (*n*) (RR [95% CI])
Primary analysis	−27.50 (−42.20, −12.81)	−93.15 (−125.29, −61.00)	−138.83 (−177.57, −100.10)	−0.63 (−2.48, 1.23)	0.58 (0.33, 1.02)	0.68 (0.38, 1.19)

Sensitivity analyses						

Excluding “lower” quality studies^∗^ (Tsimoyiannis et al., 2002 [28], Chen, 2012 [20], and Inoue et al., 2012 [26])	−28.66 (−49.47, −7.85)	−79.20 (−118.09, −40.32)	−126.00 (−161.92, −90.08)	Too few studies (<2) to inform	0.41 (0.18, 0.92)	Too few studies (<2) to inform

Only monopolar electrosurgery (i.e., excluding suture ligation)	−45.15 (−58.96, −31.33)	−91.59 (−137.55, −45.62)	−93.55 (−114.08, −73.02)	Too few studies (<2) to inform	Too few studies (<2) to inform	Too few studies (<2) to inform

Excluding imputed data (Inoue et al., 2012 [26], Wilhelm, 2011 [23], and Lu et al., 2008 [24])	−27.62 (−43.84, −11.41)	−91.92 (−124.39, −59.45)	−157.39 (−196.50, −118.27)	−1.07 (−4.53, 2.40)	0.58 (0.33, 1.02)	0.68 (0.38, 1.19)

CI: confidence interval; LOS: length of stay; MD: mean difference; RR: relative risk.

^∗^Lower quality study defined as ≥4 “unclear” or one “No” listed in any risk of bias assessment category.
